# Boundaryless working hours and recovery in Germany

**DOI:** 10.1007/s00420-021-01748-1

**Published:** 2021-08-24

**Authors:** Laura Vieten, Anne Marit Wöhrmann, Alexandra Michel

**Affiliations:** 1 Federal Institute for Occupational Safety and Health (BAuA), Dortmund, Germany; 2 Department of Psychology, Heidelberg University, Heidelberg, Germany

**Keywords:** Flexible working hours, Overtime, Weekend work, Work availability, Recovery experiences

## Abstract

**Objective:**

Due to recent trends such as globalization and digitalization, more and more employees tend to have flexible working time arrangements, including boundaryless working hours. The aim of this study was to investigate the relationships of various aspects of boundaryless working hours (overtime, Sunday work, and extended work availability) with employees’ state of recovery. Besides, we examined the mediating and moderating role of recovery experiences (psychological detachment, relaxation, mastery, and control) in these relationships.

**Methods:**

We used data from 8586 employees (48% women; average age of 48 years) who took part in the 2017 BAuA-Working Time Survey, a representative study of the German working population. Regression analyses were conducted to test main effects as well as mediation and moderation.

**Results:**

Overtime work, Sunday work, and extended work availability were negatively related to state of recovery. Psychological detachment mediated these relationships. Furthermore, we found that relaxation and control mediated the association between extended work availability and state of recovery. However, no relevant moderating effects were found.

**Conclusions:**

Altogether, our findings indicate that various aspects of boundaryless working hours pose a risk to employees’ state of recovery and that especially psychological detachment is a potential mechanism in these relationships. In addition, the results suggest that a high level of recovery experiences cannot attenuate these negative relationships in leisure time. Therefore, employers and employees alike should try to avoid or minimize boundaryless working hours.

## Introduction

Recent trends such as globalization and digitalization have contributed to the emergence of a 24/7 economy, in which traditional nine-to-five jobs are increasingly replaced by more flexible working time arrangements (Amlinger-Chatterjee 2016; Fagan et al. 2012). For example, in 2015, almost half of the employees in Germany regularly worked on weekends (48%), 48 percent of employees were at least sometimes requested to come into work at short notice, and 24 percent worked more than ten hours a day at least once a month (Eurofound 2016). The relevance of flexible working hours has presumably further intensified, not least due to the SARS-CoV-2 pandemic. Although flexible working hours may benefit employers and employees alike (e.g., by providing more autonomy; Seitz and Rigotti 2018), many scientists warn against negative consequences such as impaired recovery being pivotal for employees’ well-being, health, and performance (e.g., Amlinger-Chatterjee 2016; Tucker and Folkard 2012). The latter is mainly because flexible working hours bear the risk of extended and boundaryless working hours.

According to boundary theory (Ashforth et al. 2000; Nippert-Eng 1996), individuals create boundaries between work and private life. We define boundaryless working hours as working hours that lie outside the contractually defined working hours or exceed them, thereby increasingly blurring these boundaries. Studies have shown that various aspects of boundaryless working hours, such as overtime work (e.g., Jansen et al. 2003) or extended work availability (e.g., Rau and Göllner 2019), are negatively related to employees’ recovery. However, only few studies investigate the impact of other aspects of boundaryless working hours on recovery. For example, little is known about the effect of work on typically work-free days, such as Sunday work in Germany, on employees’ recovery.

Moreover, there is little research on the mediating process through which boundaryless working hours negatively influence employees’ state of recovery. However, identifying relevant mediators is important to understand the mechanism that causes the negative effects theoretically. This, in turn, will enable researchers and practitioners to design strategies to cope better with boundaryless working hours. In this study, we focus on recovery experiences (Sonnentag and Fritz 2007) as possible important mediators because they are closely related to employees’ state of recovery (Steed et al. 2019). Evidence from the few studies available so far suggests that they might act as mediators (e.g., Dettmers et al. 2016b; Dettmers 2017). In addition, the stressor-detachment model (Sonnentag 2011; Sonnentag and Fritz 2015) states that psychological detachment functions as a mediator in the relationship between job stress and strain, and impaired well-being.

Furthermore, the moderating role of recovery experiences such as psychological detachment, relaxation, mastery, and control (Sonnentag and Fritz 2007) in the relationship between boundaryless working hours and state of recovery has hardly been studied. For instance, we are only aware of one study, which has investigated a moderating effect of psychological detachment in the context of working hours and well-being (Lu and Chou 2020). We believe, however, that the investigation of moderating effects is important to find approaches to mitigate the adverse effects of boundaryless working hours, which cannot always be avoided.

In this study, we aim to address this lack of research and further examine the relationship between boundaryless working hours and employees’ state of recovery. We also want to investigate whether recovery experiences play a mediating and moderating role in these relationships. To this end, we consider three aspects of boundaryless working hours: overtime, Sunday work, and extended work availability. As far as we know, this study is the first to investigate the potential mediating and moderating effects of all four recovery experiences highlighted by Sonnentag and Fritz (2007) in the context of boundaryless working hours. Besides, this is one of the first research articles on boundaryless work and recovery using data from a large national survey of employees in Germany, including both white- and blue-collar workers from all industries and regions. Thus, we provide results that are generalizable to the majority of the German workforce.

### Boundaryless working hours and recovery

Work schedules are often characterized in terms of three dimensions of working time: (1) duration of working time, (2) position of working time within a day and a week, and (3) flexibility of working time in terms of (short-term/unpredictable) changes of working hours (e.g., Janssen and Nachreiner 2004; Piasna 2018). These three dimensions can also characterize boundaryless working hours. To address all three working time dimensions, in this study, we address overtime referring to boundaryless working hours in terms of duration, Sunday work relating to position, and extended work availability as an aspect of flexibility.

When employees are exposed to high work demands or stressors, several physiological stress systems are activated, including the sympathetic-adrenal-medullary system and the hypothalamic–pituitary–adrenal system (Geurts and Sonnentag 2006; Sonnentag and Geurts 2009). This leads to cardiovascular and neuroendocrine responses, such as increased heart rate or cortisol excretion. Recovery is defined as a psychophysiological unwinding and restoration “process during which [these] individual functional systems […] return to their prestressor levels” (Sonnentag and Fritz 2007, p. 205; see also Meijman and Mulder 1998). It is often reflected “in a restoration of impaired mood and action prerequisites” as well as “in a decrease in physiological strain indicators” (Sonnentag and Fritz 2007, p. 205). According to Sonnentag and Geurts (2009), the phenomenon of recovery not only encompasses recovery as a process but also as an outcome. While recovery as a process refers to activities and experiences that reduce the stress level, recovery as an outcome refers to a person’s psychological or physiological state or performance reached after a successful or less successful recovery period (Sonnentag et al. 2017). In our paper, we focus on both recovery as a process by considering four recovery experiences as well as on employees’ state of recovery. Regarding employees’ state of recovery, we refer to employees’ psychological state, more specifically their self-assessed level of feeling recovered.

It can be assumed that working time arrangements are closely related to employees' recovery. Working hours determine employees’ rest periods and, therefore, the time available for recovery. Boundaryless working hours usually imply prolonged working hours and, therefore, extended exposure to work demands resulting in an increased need for recovery. At the same time, however, time for recovery is reduced (e.g., Caruso et al. 2006). Accordingly, a negative relationship between boundaryless working hours and employees’ state of recovery is likely. In this study, we do not solely aim to address the aspect of boundaryless working hours that relates to the duration of working time, but also those aspects relating to the position of work within a week as well as flexibility in terms of changes of working hours. Therefore, we take a more differentiated look at these three aspects of boundaryless working hours in relation to employees' state of recovery. More specifically, we focus on characteristics of boundarylessness regarding the duration of working time (overtime), the position of working time (Sunday work), and the flexibility of working time (permanent availability).

#### Overtime and state of recovery

Overtime refers to work exceeding the contractual working hours (Wöhrmann et al. 2016). Based on the effort-recovery model (Meijman and Mulder 1998), we propose that overtime is negatively related to state of recovery. The effort-recovery model (Meijman and Mulder 1998) assumes that work demands require employees to spend effort, leading to load reactions such as work-related fatigue or physiological activation (see also Binnewies and Sonnentag 2008). In addition, it assumes that recovery occurs automatically and “individual functional systems that have been called upon during a stressful experience return to their prestressor levels” (Sonnentag and Fritz 2007, p. 205; Meijman and Mulder 1998) when a person is no longer exposed to work or similar demands. Since overtime implies an extension of working hours, the exposure to work demands is prolonged (Caruso et al. 2006), load reactions may accumulate, and the need for recovery may increase. Simultaneously, overtime implies that the functional systems active during regular working hours remain active for a prolonged time. Thus, recovery is not possible during overtime according to the effort-recovery model (Meijman and Mulder 1998).

Past research found significant relationships between overtime or long working hours and the need for recovery (e.g., Kinnunen et al. 2011; Mohren et al. 2010). For instance, in a large cross-sectional study, Jansen et al. (2003) found that frequent overtime workers reported higher needs for recovery. Thus, based on the effort-recovery model (Meijman and Mulder 1998) and previous empirical findings, we propose the following hypothesis:

Hypothesis 1: Overtime is negatively related to state of recovery.

#### Sunday work and state of recovery

The second aspect of boundaryless working hours investigated in this study is Sunday work. Based on the conservation of resources (COR) theory (Hobfoll 1989, 1998), we propose that Sunday work is negatively related to state of recovery. COR theory (Hobfoll 1989, 1998) states that people generally strive to retain, protect, and build resources, which are defined as “those objects, personal characteristics, conditions, or energies that are valued by the individual or that serve as a means for attainment of these” (Hobfoll 1989, p. 516). According to this theory and regarding the work context, stress occurs when work demands threaten or deplete a person’s resources (Siltaloppi et al. 2009). Recovery occurs when employees restore threatened or lost resources and gain new ones in off-job time. However, a gain of resources usually does not happen automatically but requires the investment of some other resources (Binnewies and Sonnentag 2008; Hobfoll 1989).

Some European countries, such as Austria, Germany, and Norway, legally regulate Sunday work. In Germany, for example, Sunday work is only permitted in exceptional cases, for example, in hospitals or on farms. Hence, Sunday is a work-free day for most German employees and therefore, it is usually considered as a day for social and family activities and rest (Wirtz et al. 2011). Following our definition of boundaryless working hours, we define Sunday work as not contractually agreed work performed on Sundays. Social activities provide the opportunity for social support, which according to COR theory (Hobfoll 1989, 1998), is an essential resource that is not only important in itself but also because it “contribute[s] to a maintenance of strong resource reservoirs” (Hobfoll 2001, p. 349). Past research indicates that Sunday workers cannot fully compensate for the socially valuable time lost because of Sunday work, not even by taking time off on another weekday (e.g., Barnes et al. 2006; Bittman 2005). Since Sunday workers thus have fewer opportunities to create resources, we assume in line with COR theory (Hobfoll 1989, 1998) that Sunday work is negatively related to state of recovery.

Research on the effects of Sunday work on employees’ recovery has been relatively scarce. However, few studies found associations of weekend work with sleep problems, fatigue, and health problems (Karhula et al. 2020; Wirtz et al. 2011). Altogether, our theoretical thoughts on the qualitative importance of Sundays for resource gain lead us to the following assumption:

Hypothesis 2: Sunday work is negatively related to state of recovery.

#### Extended work availability and state of recovery

We examine extended work availability, defined as “a condition in which employees formally have off-job time but are flexibly accessible to supervisors, coworkers, or customers and are explicitly or implicitly required to respond to work requests” (Dettmers et al. 2016b, p. 106) as a third aspect of boundaryless working hours. In this paper, we focus on a core feature of extended work availability, namely contacting frequency. It describes how often employees are contacted for work issues in private life.

Based on boundary theory (Ashforth et al. 2000; Nippert-Eng 1996) and the effort-recovery model (Meijman and Mulder 1998), we assume that extended work availability is negatively related to state of recovery. Boundary theory (Ashforth et al. 2000; Nippert-Eng 1996) states that people create and maintain boundaries between various life domains such as work and private life to structure their environment. It also assumes that behavioral, temporal, physical, and communicative tactics are used to create these boundaries, which can differ in their degree of flexibility and permeability. “Flexibility is the degree to which […] boundaries are pliable” (Ashforth et al. 2000, p. 474), while permeability is the degree to which elements are allowed to enter from one domain into another (Ashforth et al. 2000). From the perspective of boundary theory (Ashforth et al. 2000; Nippert-Eng 1996), extended work availability implies that boundaries between work and private life are permeable because elements from the work domain enter the private life domain. This also means that work-related thoughts or worries can spill over (e.g., Hahn and Dormann 2013; Ilies et al. 2009).

Thinking about work in private life can be reinforced by the unpredictability of being contacted for work-related reasons, which is a unique characteristic of extended work availability. Following Dettmers et al. (2016b), we assume this unpredictability to be associated with permanent activation and anticipatory stress because employees mentally and behaviorally prepare for incoming calls and possibly resulting work. Hence, extended work availability causes the functional systems activated during regular working hours to remain active. Thus, according to the effort-recovery model (Meijman and Mulder 1998), recovery cannot occur.

Recent studies support the assumption of a negative relationship between extended work availability and state of recovery (e.g., Gombert et al. 2018; Rau and Göllner 2019). Altogether, based on the theoretical assumptions made and these empirical findings, we hypothesize:

Hypothesis 3: Extended work availability is negatively related to state of recovery.

### Recovery experiences as mediators

Recovery experiences might mediate the relationships of boundaryless working hours and state of recovery. Four recovery experiences can be differentiated: psychological detachment, relaxation, mastery experiences, and control during leisure time (Sonnentag and Fritz 2007). Psychological detachment can be defined as switching off during off-job time or mentally disengaging oneself from work (Sonnentag and Bayer 2005; Sonnentag and Fritz 2007). Relaxation “is characterized by a state of low activation and increased positive affect” (Sonnentag and Fritz 2007, p. 206; Stone et al. 1995). Mastery experiences relate to “off-job activities that distract from the job by providing challenging experiences and learning opportunities in other domains” and thereby “offer opportunities for experiencing competence and proficiency” (Sonnentag and Fritz 2007, p. 206). Finally, control during leisure time allows employees to choose which activity to pursue during their free time and when and how to pursue it (Sonnentag and Fritz 2007).

To explain how these four recovery experiences help recovery to occur, Sonnentag and Fritz (2007) refer to the effort-recovery model (Meijman and Mulder 1998) and the COR theory (Hobfoll 1989, 1998). These two theories describe two complementary processes by which recovery occurs (Sonnentag and Fritz 2007). According to the effort-recovery model (Meijman and Mulder 1998), psychological detachment and relaxation may help recovery because they increase the chance that no further demands tax the functional systems used during work (Sonnentag and Fritz 2007) and recovery thus occurs automatically. In line with COR theory (Hobfoll 1989, 1998), it is helpful to gain new resources during leisure time, such as energy, self-efficacy, or positive mood, to restore threatened and lost resources (Sonnentag and Fritz 2007). Hence, mastery experiences and control during leisure time are helpful as they offer the opportunity to gain new resources and restore threatened ones. To date, the direct relationships of the four recovery experiences with state of recovery or need for recovery are well established (for an overview, see Steed et al. 2019).

In addition, we assume negative relationships between boundaryless working hours and recovery experiences. As boundaryless working hours imply the occupation with work tasks during times originally or traditionally intended for free time, we assume that boundaryless working hours and psychological detachment are negatively related. Thus, they make psychological detachment impossible during these times. Furthermore, boundaryless working hours may increase the risk of rumination about work during leisure time (Cropley and Millward Purvis 2003) and thus hinder psychological detachment. In line with this assumption, several studies found negative associations of overtime work or the length of working hours (for an overview, see Wendsche and Lohmann-Haislah 2017), work on weekends (Weigelt and Syrek 2017), extended work availability (e.g., Dettmers 2017; Dettmers et al. 2016a), and working boundlessly in time (i.e., working hours that are very spread out across the working day and week; Mellner et al. 2016) with psychological detachment.

Similar theorizing applies to relaxation. Since exposure to (work) demands is related to perseverative cognition and thus prolonged activation (Brosschot et al. 2005), we assume that boundaryless working hours are associated with problems in relaxation during leisure time (Sonnentag and Fritz 2007). Accordingly, some studies have found negative relationships of overtime work or working hours (e.g., Kinnunen et al. 2011; Sonnentag and Fritz 2007) and work-related task during leisure time (e.g., ten Brummelhuis and Bakker 2012) with relaxation.

We also expect a negative relationship between boundaryless working hours and mastery. Our expectation is based on the assumption that work demands lead to fatigue (Zohar et al. 2003), challenging to invest the effort and self-regulation (Muraven et al. 1998) required for mastery experiences (Sonnentag and Fritz 2007).

Finally, we expect a negative relationship between boundaryless working hours and control during leisure time. Boundaryless working hours often limit or fragment leisure time and thus employees’ control over it (Sonnentag and Fritz 2007). Moreover, work demands are often associated with rumination in leisure time (Cropley and Millward Purvis 2003) causing the feeling of having little control (Kinnunen et al. 2011). Previous research on the relationship between boundaryless working hours and employees’ control during leisure time has already found negative correlations (Dettmers et al. 2016a; Kinnunen et al. 2011).

Based on the theoretical assumptions and empirical findings described above supporting negative relationships between boundaryless working hours and recovery experiences as well as positive relationships between recovery experiences and state of recovery, we assume that recovery experiences mediate the relationship between boundaryless working hours and employees’ state of recovery. The assumption of psychological detachment as a mediator is also one of the main assumptions of the stressor-detachment model (Sonnentag 2011; Sonnentag and Fritz 2015), which states that psychological detachment functions as a mediator in the relationship between job stress and strain, and impaired well-being. To extend this model, we assume that the recovery experiences of relaxation, mastery, and control also act as mediators and propose the following hypothesis:

Hypothesis 4: The negative relationships between boundaryless working hours and state of recovery are mediated by (a) psychological detachment, (b) relaxation, (c) mastery experiences, (d) control during non-work times.

### Recovery experiences as moderators

In addition to their mediating role, recovery experiences could also act as moderators in the relationship between boundaryless working hours and state of recovery. In line with this, Sonnentag and Fritz (2007) suggested that recovery experiences “might be conceptualized as […] moderator[s] in the relation between job stressors and impaired wellbeing with poor recovery experiences increasing the association between job stressors and poor wellbeing” (p. 218). Furthermore, regarding psychological detachment, the stressor-detachment model (Sonnentag 2011) describes psychological detachment as both a mediator and a moderator in the relationship between job stressors and strain. Its moderating effect is based on the assumption that job stressors affect employees during and after work, for example, by remembering a stressful work situation in leisure time. Psychological detachment, however, implies disengaging oneself mentally from work, and therefore it enables recovery and reduces strain. Thus, psychological detachment can attenuate the negative relationship between job stressors and strain (Sonnentag 2011; Sonnentag and Fritz 2015).

Several studies have investigated and found the buffer-function of recovery experiences (e.g., Kinnunen et al. 2010; Sonnentag et al. 2013). For instance, Siltaloppi et al. (2009) found that psychological detachment and mastery attenuated the negative relationship between job control and need for recovery, while relaxation attenuated the positive relationship between time demands and job exhaustion. However, we are only aware of one study investigating a moderating effect of recovery experiences in the context of (boundaryless) working hours (Lu and Chou 2020). The authors find psychological detachment to moderate the negative effect of working hours on work engagement and job performance, measured six months later.

As our Hypotheses 1–3 indicate, we expect boundaryless working hours to impede employees’ state of recovery. Following the suggestion of Sonnentag and Fritz (2007) that recovery experiences might moderate the relation of job stressors and well-being, the stressor-detachment model (Sonnentag 2011) assuming psychological detachment as moderator, and previous research indicating moderating effects of recovery experiences, it could be assumed that recovery experiences also moderate the relation between boundaryless working hours and state of recovery.

For our assumption of moderating effects of recovery experiences, it is important to know that we assume recovery experiences to entail dispositional aspects. We base this assumption on previous research that has shown that individuals exhibit substantial consistency in their recovery experiences over time (for an overview, see Steed et al. 2019). These findings indicate that “certain individuals may be more or less prone to engage in recovery experiences due to personality factors or routines, regardless of situational or contextual factors” (Steed et al. 2019, p. 27). Therefore, we additionally assume that that individual differences in recovery experiences can influence the relationship between boundaryless working hours and state of recovery.

Boundaryless working hours imply that time for recovery is reduced and fragmented. However, the previous assumptions also imply that a high quality of the remaining leisure time, that is, by engaging in recovery experiences, can compensate for the reduced recovery time. For instance, if employees are better able to detach or relax in the remaining leisure time after working overtime, on Sundays, or after being contacted, work demands in terms of work-related thoughts are present for a shorter time allowing these employees to recover more quickly. In addition, these employees might gain more resources in the remaining leisure time, for example, through engaging in mastery experiences, which helps recovery. Employees with a high degree of control can also react more flexible to boundaryless working hours because they can more easily postpone potential recovery activities to the remaining free time than employees with a low degree of control. Thus, we expect employees with high levels of psychological detachment, relaxation, mastery, or control during leisure time to report higher states of recovery than employees with poor recovery experiences when confronted with a high amount of boundaryless working hours. Thus, we hypothesize:

Hypothesis 5: The negative relationships between boundaryless working hours and state of recovery are moderated (attenuated) by (a) psychological detachment, (b) relaxation, (c) mastery experiences, (d) control during non-work times.

## Methods

### Sample and procedure

We used data from the second wave of the BAuA-Working Time Survey, which took place in 2017 (for a detailed description of the survey, its sample, and methodology, see Häring et al. 2018; Wöhrmann et al. 2021), and which is representative of a large part of the German working population. About 10,500 individuals were asked about their working conditions focusing on aspects related to working time, as well as their health and well-being. In addition, this survey wave contained a special module on recovery, including several questions about participants’ state of recovery and their recovery experiences. Data were collected utilizing completely standardized computer-assisted telephone interviews (CATI), which lasted on average 35 min.

For the present study, we restricted the sample to employees aged 15–65 years. Thus, our final sample consisted of 8586 employees (48% women; average age of 48.49 years, *SD* = 10.10). One-third of these participants (34%) worked in the public sector, 29 percent in the service sector, 21 percent in the industrial sector, 7 percent in the craft sector, and 10 percent worked in a different area or could not classify their employer. Further information on the sample is provided in Table 1.

**Table 1:** Descriptive statistics and correlations for study variables

Variable	*n *^*a*^	% ^*a*^	1	2	3	4	5	6	7	8	9	10	11	12	13	14	15
1. Gender	4147	48	–														
2. Education	4571	53	− 0.07**	–													
3. Child in household	2816	33	− 0.04**	0.07**	–												
4. Full-time job	6398	75	− 0.47**	0.07**	− 0.10**	–											
5. Regular day work	7036	82	0.03*	0.17**	0.04**	− 0.02	–										
6. Workload	995	12	0.05**	− 0.01	− 0.01	− 0.03*	− 0.05**	–									
7. Mental or physical activity at work	3445	40	− 0.02	− 0.35**	− 0.06**	− 0.04**	− 0.33**	0.02	–								
	*M*	*SD*															
8. Age	48.49	10.10	0.07**	0.03*	− 0.29**	− 0.04**	0.01	− 0.03	− 0.01	–							
9. Overtime	4.19	5.67	− 0.12**	0.12**	0.02	0.13**	− 0.03	0.10**	− 0.02	− 0.04**	–						
10. Work on Sundays	0.47	1.01	0.02	− 0.02	− 0.02	0.00	− 0.32**	0.07**	0.19**	− 0.03*	0.26**	–					
11. Extended work availability	2.27	0.88	0.01	0.06**	0.05**	− 0.01	− 0.08**	0.12**	0.06**	− 0.06**	0.26**	0.25**	–				
12. Psychological detachment	3.48	1.24	0.02	− 0.12**	0.00	− 0.03*	− 0.01	− 0.18**	0.08**	0.01	− 0.16**	− 0.11**	− 0.27**	–			
13. Relaxation	4.24	0.93	− 0.03	− 0.03	− 0.09**	0.05**	− 0.01	− 0.14**	0.03	0.08**	− 0.06**	− 0.04**	− 0.13**	0.31**	–		
14. Mastery	3.44	1.06	− 0.07**	0.04**	− 0.01	0.03	0.02	− 0.07**	− 0.01	− 0.01	0.00	− 0.01	0.01	0.13**	0.30**	–	
15. Control	4.32	0.97	− 0.02	− 0.09**	− 0.21**	0.08**	− 0.05**	− 0.11**	0.09**	0.07**	− 0.02	− 0.01	− 0.08**	0.19**	0.42**	0.18**	–
16. State of recovery	3.97	0.86	− 0.01	0.03*	0.03	− 0.06**	0.14**	− 0.27**	− 0.12**	0.07**	− 0.15**	− 0.17**	− 0.19**	0.25**	0.27**	0.12**	0.18**

*n* = 7862–8586. gender: 1 = female; education: 1 = high level; child in household: 1 = yes; full-time job: 1 = full-time job; regular day work: 1 = working hours usually between 7 am and 7 pm; workload: 1 = overchallenged; mental or physical activity at work: 1 = mainly physically active or equally mentally and physically active

^a^ Reflects the number or percentage of participants with the value 1

^*^p < .01. ^*^^*^p < .001

### Measures

#### Boundaryless working hours

To measure boundaryless working hours, we considered three aspects of blurred boundaries related to the duration, position, and flexibility of working time, namely overtime, Sunday work, and extended work availability.

Working *overtime* was calculated as the difference between employees’ actual and contractual weekly working hours*.* Actual weekly working hours were assessed with the question: “How many hours do you actually work per week, on average in this occupational activity, including regular overtime work, extra work, emergency service, etc.?” Contractual weekly working hours were measured with the question: “What are the weekly working hours in your occupational activity contractually agreed with your employer, excluding overtime?” We excluded employees with a negative value in our analyses because we assume that working fewer hours than contractually agreed is qualitatively different from working overtime.

*Sunday work* was measured with the question: “Do you work—even if only occasionally—on Sundays and public holidays?” Participants who affirmed this question were then asked, “How many Sundays and public holidays a month do you work, on average?”, while participants who denied this question were directly coded 0.

We assessed *extended work availability* with the question: “How often are you contacted by employees, colleagues, supervisors, or customers in your private life?” Response categories were *never* (1), *rarely* (2), *sometimes* (3), or *often* (4).

#### State of recovery

Three translated and adapted items from the intershift recovery subscale of the Occupational Fatigue Exhaustion/Recovery Scale by Winwood et al. (2005, 2006) were used to measure employees’ state of recovery. Intershift recovery is defined as the “extent to which recovery is achieved from one work shift to the next” (Winwood et al. 2005, p. 598). One sample item was: “Before work I normally feel fully recovered.” In addition, the following item, which refers to a longer possible recovery period between two work shifts, was used: “After the weekend or after my days off, I normally feel recovered.” Thus, we measured employees’ state of recovery with four items with possible responses on a five-point Likert scale from 1 (*strongly disagree*) to 5 (*strongly agree*). Regarding reliability, Cronbach’s alpha in the sample was 0.67, while McDonald’s omega was 0.69.

#### Recovery experiences

Each of the four recovery experiences was assessed with one item of Sonnentag and Fritz’s (2007) Recovery Experience Questionnaire. These were: “In my free time I forget about work” (*psychological detachment*), “In my free time I do relaxing things” (*relaxation*), “In my free time I do things that challenge me” (*mastery experiences*), and “In my free time I feel like I can decide for myself what to do” (*control*). Participants could respond on a five-point Likert scale from 1 (*strongly disagree*) to 5 (*strongly agree*).

#### Control variables

This study aimed to determine the effects of boundaryless working hours on employees’ recovery. To ensure that our results will be due to the boundarylessness of working hours and not to other working (time) conditions or socio-demographic aspects, we control for different sets of antecedents of recovery from work.

Based on theoretical assumptions (see above) and previous studies (e.g., Arlinghaus and Nachreiner 2014; Kiss et al. 2008), we assume that working time arrangements are related to employees’ recovery. However, since we aim to examine the effect of boundarylessness working hours beyond working hours per se, we controlled for employment status as part-time or full-time (0 = part-time, 1 = full-time) and regular day work (0 = working hours usually not between 7 am and 7 pm, 1 = working hours usually between 7 am and 7 pm).

Regarding further working conditions, we included demands regarding the workload (0 = underchallenged or generally feels up to the demands, 1 = overchallenged) as a control variable in our analyses because meta-analyses (e.g., Bennett et al. 2018; Steed et al. 2019) show that individuals with a higher workload report poorer recovery experiences and state of recovery. In addition, we controlled for employees’ type of work (0 = mainly mentally active, 1 = mainly physically active or equally mentally and physically active) because studies have indicated that these may relate to both employees’ working hours and recovery (e.g., Arlinghaus and Nachreiner 2014).

Not only work demands but also home demands can affect recovery from work (Steed et al. 2019). To account for the role of employees’ family situation regarding the opportunities to recover in non-work time (e.g., Virtanen et al. 2020), we controlled for the existence of underage children in the household (0 = no, 1 = yes). According to findings from several studies, further socio-demographic factors such as gender, age, and level of education are relevant in the context of recovery (e.g., Jansen et al. 2002; Kiss et al. 2008; Sonnentag and Zijlstra 2006). Therefore, we included gender (0 = male, 1 = female), age (in years), and level of education according to ISCED 2011 (UNESCO Institute for Statistics 2012; 0 = low or medium level of education, 1 = high level of education) as control variables in our analyses.

### Statistical analyses

We tested Hypotheses 1–3 and Hypothesis 5 using standard hierarchical multiple regression analyses in SPSS 26.0. To facilitate the interpretability of the coefficients, we mean-centered the variables of the three aspects of boundaryless working hours and the four recovery experiences before the analyses. We conducted separate regression analyses for each of the four recovery experiences using the following procedure: In the first step, we entered the eight control variables. In the second step, we included overtime work, Sunday work, and extended work availability to test the main effects (Hypotheses 1–3). In the third step, the specific recovery experience was entered. Finally, in the fourth step, we entered the three interaction terms to test the interaction effects (Hypothesis 5). In addition, we performed a regression analysis with all four recovery experiences to examine if these operated independently. Given the large sample size, a 1% alpha level was applied in this study for all significance tests.

Hypothesis 4 was tested with regression analyses using Model 4 of Hayes’s (2018) PROCESS macro in SPSS 26.0. Ninety-nine percent confidence intervals were generated for the indirect effects of overtime work, Sunday work, and extended work availability mediated by recovery experiences. We used bootstrapping with 10,000 draws. Again, a separate analysis was conducted for each of the four recovery experiences. Furthermore, we used the same control variables as in the moderation analyses.

## Results

### Descriptive results

Descriptive statistics, including bivariate correlations for all study variables, are shown in Table 1. As expected, all three aspects of boundaryless working hours showed significant negative correlations with employees’ state of recovery. Boundaryless working hours were also negative correlated with psychological detachment and relaxation. Regarding mastery experiences, none of the three aspects of boundaryless working hours showed a significant correlation, and regarding control during leisure time, one significant negative correlation with extended work availability was observed. In addition, all four recovery experiences showed significant positive correlations with state of recovery.

### Results of hypotheses testing

#### Main effects results

After controlling for several socio-demographic factors and working conditions, overtime (β =  − 0.079, *p* < 0.001), Sunday work (β =  − 0.070, *p* < 0.001), and extended work availability (β =  − 0.119, *p* < 0.001) were significantly negatively related to state of recovery. These results provide support for Hypothesis 1–3, which stated negative relationships of boundaryless working hours and state of recovery.

#### Mediation results

Table 2 shows the results from mediation analyses. Significance of relationships was assumed if confidence intervals did not include zero. All three aspects of boundaryless working hours and all four recovery experiences showed significant direct associations with state of recovery. Regarding the relationships of boundaryless working hours and recovery experiences, overtime work, Sunday work, and extended work availability showed significant negative relationships with psychological detachment. However, mastery was not significantly related to any of these three aspects of boundaryless working hours. With regard to relaxation and control, only extended work availability showed significant negative associations with these recovery experiences.

**Table 2:** Results from regression analyses with recovery experiences as mediators of the relationship between boundaryless working hours (independent variables) and state of recovery (dependent variable)

	Mediator: Detachment				Mediator: Relaxation				Mediator: Mastery				Mediator: Control			
	*b*	*SE*	LLCI	ULCI	*b*	*SE*	LLCI	ULCI	*b*	*SE*	LLCI	ULCI	*b*	*SE*	LLCI	ULCI
Direct effects on recovery experience																
Direct effect of overtime work	− 0.016	0.003	− 0.022	− 0.009	− 0.002	0.002	− 0.007	0.003	− 0.003	0.002	− 0.009	0.003	0.002	0.002	− 0.004	0.007
Direct effect of work on Sundays	− 0.044	0.015	− 0.083	− 0.005	− 0.014	0.012	− 0.045	0.016	− 0.006	0.014	− 0.041	0.030	− 0.020	0.012	− 0.051	0.012
Direct effect of extended work availability	− 0.313	0.016	− 0.354	− 0.272	− 0.114	0.012	− 0.146	− 0.082	0.027	0.014	− 0.010	0.064	− 0.065	0.013	− 0.098	− 0.032
Direct effects on state of recovery																
Direct effect of overtime work	− 0.010	0.002	− 0.015	− 0.006	− 0.012	0.002	− 0.016	− 0.007	− 0.013	0.002	− 0.017	− 0.008	− 0.013	0.002	− 0.017	− 0.008
Direct effect of work on Sundays	− 0.057	0.010	− 0.083	− 0.030	− 0.059	0.010	− 0.085	− 0.033	− 0.062	0.010	− 0.089	− 0.035	− 0.059	0.010	− 0.086	− 0.033
Direct effect of extended work availability	− 0.078	0.011	− 0.106	− 0.049	− 0.092	0.011	− 0.119	− 0.064	− 0.117	0.011	− 0.145	− 0.089	− 0.107	0.011	− 0.135	− 0.079
Direct effect of recovery experience	0.123	0.008	0.104	0.143	0.218	0.010	0.193	0.244	0.085	0.009	0.063	0.108	0.150	0.010	0.125	0.175
Indirect effects on state of recovery via recovery experience																
Indirect effect of overtime work	− 0.002	0.000	− 0.003	− 0.001	− 0.001	0.001	− 0.002	0.001	− 0.000	0.000	− 0.001	0.000	0.000	0.000	− 0.001	0.001
Indirect effect of work on Sundays	− 0.006	0.002	− 0.011	− 0.000	− 0.003	0.003	− 0.010	0.004	− 0.001	0.001	− 0.004	0.003	− 0.003	0.002	− 0.008	0.002
Indirect effect of extended work availability	− 0.039	0.003	− 0.048	− 0.030	− 0.025	0.003	− 0.033	− 0.017	0.002	0.001	− 0.001	0.006	− 0.010	0.002	− 0.015	− 0.005
Total effects on state of recovery																
Total effect of overtime work	− 0.012	0.002	− 0.017	− 0.008	− 0.012	0.002	− 0.017	− 0.008	− 0.013	0.002	− 0.017	− 0.008	− 0.012	0.002	− 0.017	− 0.008
Total effect of work on Sundays	− 0.062	0.010	− 0.089	− 0.035	− 0.062	0.010	− 0.089	− 0.035	− 0.063	0.010	− 0.089	− 0.036	− 0.062	0.010	− 0.089	− 0.035
Total effect of extended work availability	− 0.116	0.011	− 0.145	− 0.088	− 0.117	0.011	− 0.145	− 0.088	− 0.115	0.011	− 0.143	− 0.087	− 0.117	0.011	− 0.145	− 0.088

*n* = 7755–7760. LLCI = lower limit of the 99% confidence interval; ULCI = upper limit of the 99% confidence interval. All analyses are adjusted for gender, age, education, child in household, full-time job, regular day work, demands regarding the workload, and mental or physical activity at work

These findings were also reflected in the results of the indirect effects of overtime work, Sunday work, and extended work availability on state of recovery. Regarding the analyses with psychological detachment as a mediator, none of the confidence intervals for the indirect effects included zero. In the analyses with relaxation, mastery, or control as a mediator, only the indirect effects of extended work availability via relaxation and via control were significant. Thus, our results support Hypothesis 4a, which assumed a mediating effect of psychological detachment on the relationship between boundaryless working hours and state of recovery. In addition, they partially confirmed Hypotheses 4b and 4d, namely regarding the mediating effect of relaxation and control during leisure time for the relationship between extended work availability and state of recovery.

#### Moderation results

**Fig. 1:**
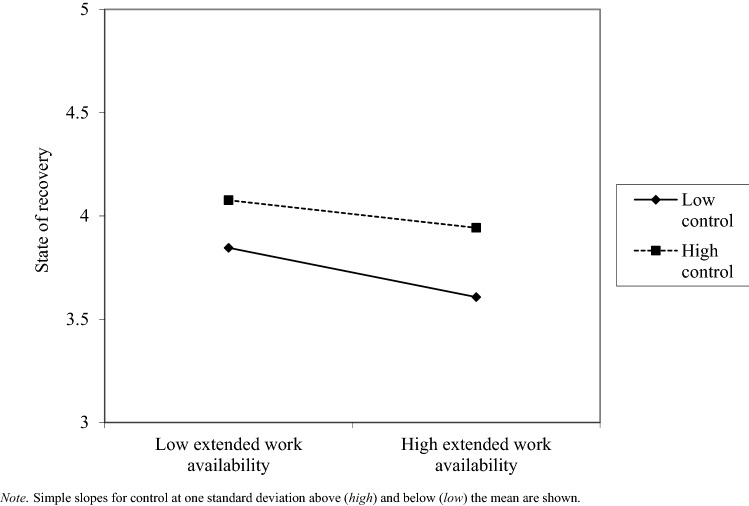
Moderating effect from control on the relationship of extended work availability (independent variable) and state of recovery (dependent variable)

The results of the hierarchical multiple regression analyses are summarized in Table 3. Across the regression models with detachment, relaxation, and mastery as moderators, none of the interaction terms was a significant predictor of state of recovery. Comparisons of the adjusted *R*^*2*^ of the models with and without interaction terms indicated that the interaction terms did not significantly contribute to the explained variance (Δ *R*^*2*^: 0.000–0.001). Regarding control, we found a significant moderating effect in the relationship between extended work availability and state of recovery (β = 0.031, *p* < 0.01). As Fig. 1 shows, the negative effect of extended work availability seems weaker when employees have a high level of control, supporting our assumption of a buffering effect. However, the amount of explained variance in employees’ state of recovery, which also includes the contributions of the interactions of overtime and Sunday work with control, is negligible (Δ *R*^*2*^: 0.001). In addition, in the regression model with all four recovery experiences, none of the interaction terms was significant. In total, we thus found no support for relevant moderating effects proposed in Hypothesis 5.

**Table 3:** Results from regression analyses with interaction effects of recovery experiences in the relationship between boundaryless working hours (independent variables) and state of recovery (dependent variable)

	Moderator(s)				
	Detachment	Relaxation	Mastery	Control	All recovery experiences
Independent Variables	β	β	β	β	β
Step 1: Control variables					
Gender	− 0.058**	− 0.052**	− 0.046**	− 0.054**	− 0.053**
Age	0.054**	0.042**	0.057**	0.053**	0.045**
Education	0.024	0.014	0.008	0.018	0.023
Child in household	0.026	0.045**	0.034*	0.063**	0.054**
Full-time job	− 0.078**	− 0.087**	− 0.078**	− 0.089**	− 0.089**
Regular day work	0.065**	0.066**	0.062**	0.070**	0.069**
Workload	− 0.209**	− 0.209**	− 0.229**	− 0.218**	− 0.190**
Mental or physical activity at work	− 0.078**	− 0.074**	− 0.068**	− 0.078**	− 0.086**
Total *R*^*2*^	0.104	0.104	0.104	0.104	0.104
Adjusted *R*^*2*^	0.103	0.103	0.103	0.103	0.103
Δ *R*^*2*^	0.104**	0.104**	0.104**	0.104**	0.104**
Step 2: Boundaryless working hours					
Overtime	− 0.072**	− 0.080**	− 0.082**	− 0.084**	− 0.076**
Work on Sundays	− 0.064**	− 0.068**	− 0.070**	− 0.067**	− 0.063**
Extended work availability	− 0.077**	− 0.093**	− 0.120**	− 0.109**	− 0.071**
Total *R*^*2*^	0.139	0.139	0.139	0.139	0.139
Adjusted *R*^*2*^	0.138	0.138	0.138	0.138	0.138
Δ *R*^*2*^	0.035**	0.035**	0.035**	0.035**	0.035**
Step 3: Recovery experience(s)					
Detachment	0.176**				0.110**
Relaxation		0.235**			0.168**
Mastery			0.106**		0.032*
Control				0.166**	0.076**
Total *R*^*2*^	0.166	0.192	0.150	0.165	0.209
Adjusted *R*^*2*^	0.165	0.190	0.149	0.164	0.207
Δ *R*^*2*^	0.027**	0.053**	0.011**	0.026**	0.070**
Step 4: Interactions					
Overtime x detachment	− 0.014				− 0.015
Work on Sundays x detachment	− 0.002				0.002
Extended work availability x detachment	0.019				0.014
Overtime x relaxation		− 0.008			0.004
Work on Sundays x relaxation		− 0.023			− 0.020
Extended work availability x relaxation		0.015			0.006
Overtime x mastery			− 0.012		− 0.006
Work on Sundays x mastery			− 0.023		− 0.022
Extended work availability x mastery			0.005		− 0.012
Overtime x control				− 0.021	− 0.018
Work on Sundays x control				0.007	0.013
Extended work availability x control				0.031*	0.027
Total *R*^*2*^	0.167	0.192	0.151	0.166	0.211
Adjusted *R*^*2*^	0.165	0.191	0.149	0.165	0.209
Δ *R*^*2*^	0.000	0.001	0.001	0.001	0.003

*n* = 7752. R^2^ = explanation rate; Δ R^2^ = change in explanation rate in each step; gender: 1 = female; education: 1 = high level; child in household: 1 = yes; full-time job: 1 = full-time job; regular day work: 1 = working hours usually between 7 am and 7 pm; workload: 1 = overchallenged; mental or physical activity at work: 1 = mainly physically active or equally mentally and physically active. Under the respective heading of a step, all variables newly added in this step are listed. The table shows the standardized beta coefficients from the fourth and final step

^*^p < .01. ^*^^*^p < .001

## Discussion

This study extends research on the relationship between boundaryless work and recovery. We examined the effects of boundaryless working hours (overtime, Sunday work, and extended work availability) on employees’ state of recovery and whether recovery experiences mediated and moderated these effects. By using data from a representative survey of employees in Germany, we provide results that can be generalized to most of the German workforce. We found that all three aspects of boundaryless working hours were negatively related to state of recovery and that psychological detachment mediated these relationships. In addition, we found relaxation and control to mediate the relationship between extended work availability and state of recovery. However, in contrast to our hypothesis, we found no relevant moderating effects of recovery experiences.

Although some studies investigated the relationship between overtime and recovery (e.g., Jansen et al. 2003) and between extended work availability and recovery (e.g., Gombert et al. 2018), there has been little research on the relationship between work on normally work-free days, such as Sunday work in Germany, and recovery. This study found negative relations between all three aspects of boundaryless working hours and state of recovery, even after controlling for several socio-demographic factors and working conditions, including additional elements of working time arrangements, namely employment status as part-time or full-time and regular day work. Hence, we could show that each of the three dimensions (duration, position, and flexibility) of boundaryless working hours added significant unique variance in predicting employees’ state of recovery.

Furthermore, our results showed that psychological detachment mediated the negative effects of boundaryless working hours on state of recovery. These results are in line with several previous studies that also found negative relations between aspects of boundaryless working hours and psychological detachment (e.g., Dettmers 2017; Mellner et al. 2016) as well as with studies showing positive relationships between detachment and state of recovery (for an overview, see Steed et al. 2019).

However, our analyses did not indicate that mastery functioned as a mediator in the relationship between boundaryless working hours and employees’ state of recovery. This is due to the non-significant relationship between boundaryless working hours and mastery. Although contrary to our hypothesis, our results are in line with other studies, which also did not find relations between different aspects of boundaryless working hours and mastery (e.g., Burke et al. 2009; Sonnentag and Fritz 2007). A possible explanation could be that some employees are more likely to have mastery experiences when working non-boundaryless hours, while others are more likely to experience mastery when working boundaryless hours. Thus, in line with our hypothesis, some employees might indeed be fatigued due to boundaryless working hours and have difficulties investing the effort and self-regulation necessary for mastery experiences. Others, however, might not feel affected by these demands or even experience extra work as mastery (Weigelt and Syrek 2017). Some might even try to counteract the negative effects of demands by engaging in activities that provide challenging experiences (Sonnentag and Fritz 2007). Future studies should therefore look at subgroups and try to identify moderating factors.

Regarding the recovery experiences relaxation and control during leisure time, results were mixed. While these recovery experiences did not mediate the relation between overtime work or Sunday work and state of recovery, they mediated the relation between extended work availability and state of recovery. A possible explanation for the fact that relaxation and control only play a mediating role in extended work availability is its special characteristic of unpredictability. Regarding relaxation, this unpredictability may lead to permanent activation, making relaxation extremely difficult. Concerning control during leisure time, the associated unpredictability may result in a feeling of having little control.

Overall, the results of mediation analyses indicate that the relationships of all three aspects of boundaryless working hours with state of recovery are mediated by psychological detachment. Thus, they support our assumption that blurred boundaries fostering reflection or even rumination on work issues in leisure time make employees less able to detach from work. Hence, in line with the effort-recovery model (Meijman and Mulder 1998), the recovery experience psychological detachment functions as the primary mechanism explaining the negative relationship between boundaryless working hours and state of recovery. Employees with boundaryless working hours often think about work even in their off-job time and therefore fail to mentally relieve the functional systems used at work. In addition, we found the relationship between extended work availability and state of recovery to be mediated by the recovery experiences relaxation and control. Therefore, our results also indicate that an inferior recovery status due to extended work availability can result from employees being less able to relax in their leisure time and control their leisure time activities when they are contacted for work-related issues outside working hours.

Regarding moderating effects, we found no relevant interaction between any of the three aspects of boundaryless working hours and recovery experiences. This indicates that employees cannot compensate for the reduced and fragmented time for leisure and thus recreation associated with boundaryless working hours by engaging in a high level of recovery experiences during their remaining leisure time. Reduced quantity of time for recovery can therefore not be outweighed by high-quality leisure time, that is, by engaging in recovery experiences. Another explanation might be that the hypothesized interactions are more complex and involve additional variables, such as job autonomy or work-home segmentation preference. In studies on the moderating effect of psychological detachment, three-way interactions have already been found (e.g., Cheng and McCarthy 2013; Etzion et al. 1998).

In the present study, we considered both mediating and moderating effects of recovery experiences in the relationship between boundaryless working hours and employees’ state of recovery. In previous studies, recovery experiences have often been considered either as mediators (e.g., Kinnunen et al. 2011) or as moderators (e.g., Siltaloppi et al. 2009). To the best of our knowledge, there is only one study by Safstrom and Hartig (2013) that has examined whether recovery experiences can both mediate and moderate such a relationship, whereby this study only considered the recovery experience psychological detachment. However, since there is a debate in research whether recovery experiences can be both mediators and moderators at the same time and whether their function depends on certain circumstances (e.g., Sonnentag 2011; Safstrom and Hartig 2013), studies are needed that “test the mediator and the moderator hypotheses with the same data set” (Sonnentag 2011, p. 264). The present study heeds this call and finds only mediating effects of some recovery experiences in the relationship between boundaryless working hours and state of recovery but no relevant moderating effects. These findings should be considered in the design of sustainable working hours and interventions to cope with boundaryless working hours.

### Strengths, limitations, and future research

A major strength of this study is the utilization of data from a large, representative survey of the German workforce. To our knowledge, this is one of the first research articles examining recovery in such a large sample of the German working population, including employees from various occupational groups, sectors, and regions. In addition, we considered three aspects of boundaryless working hours that also often co-occur in the working world (e.g., Rau and Göllner 2019). Hence, external validity is high.

Like any study, this study has some limitations. First, given the cross-sectional nature of the data, we cannot make any causal statements about the directions of the relationships, which is particularly critical regarding our mediation analyses. Thus, we cannot rule out reverse relationships, for example, people with recovery problems are more readily available for work in off-job time. Evidence for such a relationship is, for example, provided by the longitudinal study of Thörel et al. (2021), in which a reciprocal relationship between extended work availability and psychological detachment was found. Although cross-sectional data have frequently been used in studies that investigated both the mediating and moderating role of a construct (e.g., Safstrom and Hartig 2013; Wu et al. 2012), future studies could use other research designs, such as longitudinal studies using different time lags or well-controlled intervention studies with a systematic manipulation of boundaryless working hours and/or recovery experiences.

As an additional limitation, we only used self-report measures, meaning our results might be affected by common method bias. Objective working time data, as well as physiological data for measuring state of recovery, could be used in future studies because they might provide more reliable conclusions and add new insights. However, concerning recovery experiences, there is no convincing alternative to self-reports since the participant’s subjective experience should be recorded (Sonnentag and Geurts 2009).

Another limitation relates to the German sample or rather to the socio-cultural context of this study and is especially relevant regarding the aspect of Sunday work. As mentioned above, in Germany, Sunday work is only permitted in exceptional cases and thus Sundays are usually regarded as days for social activities and rest (Wirtz et al. 2011). Future studies should examine whether the negative relationship between Sunday work and employees' state of recovery found in our study is also apparent in other cultures where Sundays are not necessarily work-free. However, since we assume that the relationship is less due to Sunday itself, but rather to its perception as a day of rest, we would speculate that similar relations are more likely to be found with work on public holidays, Fridays (e.g., in Islamic countries), or Saturdays (e.g., in Israel).

There are further limitations regarding the quality of our measurements. Due to the data collection via telephone, mostly single item measures were used in the BAuA-Working Time Survey and hence in the present study. Although single-item measures are less taxing and repetitive for participants, they also differentiate less and involve a higher risk of measurement errors than multiple-item batteries. Besides, the reliability of state of recovery measurement is slightly below the standard criterion value of 0.70. We have decided to use this measure because we were especially interested in state of recovery at the beginning of the workday. However, this content-related breadth of the construct (e.g., state of recovery after rest period between two consecutive working days vs state of recovery after time off at the weekend) could be a possible explanation for this unsatisfactory reliability. Further explanations might be the limited number of items (Vaske et al. 2017) and the use of reversed items (Suárez-Álvarez et al. 2018).

Regarding our measurements of boundaryless working hours, it is essential to note that the answer categories in the measurement of extended work availability were not very detailed and may therefore be strongly influenced by respondents’ subjective understanding. Besides, the measurement of Sunday work does not allow us to distinguish whether Sunday work is contractual work or not, although our definition of boundaryless working hours states that these lie outside the contractually defined working hours or exceed them. Thus, whether the negative relationship between Sunday work and state of recovery is due solely to boundarylessness or to Sunday work in general cannot be answered based on the results of our study and should therefore be investigated in future research.

Finally, the well-established recovery experience questionnaire might initially be designed for employees who have well-defined boundaries between work and leisure time (Safstrom and Hartig 2013). Thus, some participants might have had difficulties answering the items about their recovery experiences due to blurred boundaries and an associated vague concept of leisure time.

Although the present paper sheds light on the relationships between boundaryless working hours and recovery, some research questions remain open. For example, to better understand the effects of boundaryless working hours, future research should focus on mechanisms mediating the relationships of boundaryless working hours not only with employees’ recovery but also with their overall well-being, health, or work performance. For instance, the mediating effect of other recovery experiences such as meaning or affiliation (Newmann et al. 2014) could be examined. We believe that especially the latter could play a relevant role because our derivation of the relationship between Sunday work and state of recovery refers to social activities.

Moreover, to determine whether there are any good ways to attenuate the negative effects of boundaryless working hours on employees’ state of recovery, future studies should continue to examine moderator variables. This is particularly important because the results of our study indicate that recovery experiences do not have a relevant buffering effect. In their examination of similar relationships, previous studies found buffering effects of, for example, boundary creation (Barber and Jenkins 2014) or progress towards goal attainment during extra work (Weigelt and Syrek 2017). In addition, future studies could investigate whether recovery experiences act as moderators in interaction with other variables, such as personal characteristics.

It is also vital to examine antecedents of boundaryless working hours to address them where possible and to identify people at particular risk. For instance, future studies could look at personality traits that make individuals more prone to boundaryless working hours. Previous studies have already indicated that employees with a high career orientation (Frei and Grund 2020) or those scoring high in workaholism and associated personality traits such as perfectionism or nondelegation (Clark et al. 2016) tend to exceed their regular working hours. Moreover, results from a study by Bakker et al. (2013) indicate that the negative effects of boundaryless working hours on recovery might be stronger for employees high in workaholism.

Against the background of an increasing blurring of the boundaries between work and private life, the effects of further aspects of boundaryless working hours deserve more attention. For instance, regarding the dimension of position, evening work should also be examined. In addition, future studies should investigate the effects of boundaryless workplaces.

### Practical implications

Recovery is of great importance for employees’ well-being, health, and job performance (for an overview, see Steed et al. 2019). Well-recovered employees are therefore highly desirable for organizations. Like others before (for an overview, see Steed et al. 2019), our study shows recovery experiences to be positively related to employees’ state of recovery. At the same time, it shows that boundaryless working hours impede employees’ state of recovery via recovery experiences, especially psychological detachment. We also found that recovery experiences do not have a relevant buffering effect on the relation between boundaryless working hours and employees’ state of recovery. Thus, our findings indicate that for employees’ state of recovery, working time conditions seem to be more relevant than employees’ dispositional ability to engage in recovery experiences. Therefore, it should be in the interest of organizations to create a work(ing time) environment that enables their employees sufficient recovery.

In our study, we found that each of the three dimensions (duration, position, and flexibility) of boundaryless working hours contributes to employees’ poor state of recovery. Boundaryless working hours are often characterized by overtime work (dimension duration). We found overtime to be negatively related to employees’ recovery status and that psychological detachment is a potential mechanism. Therefore, employers and employees alike should try to avoid or minimize overtime work to help employees to detach from work in off-job time and to stay recovered and healthy. To this end, organizations could, for example, address the issues of high pace and amount of work, important determinants of boundaryless working hours (van der Hulst et al. 2006).

However, our results show that not only the duration but also the position of working hours within a week is related to employees’ state of recovery. More specifically, if employees have to work on usually work-free days, such as Sundays or public holidays, they are less able to detach from work. Thus, these days should really be work-free, because they provide employees socially valuable time and promote psychological detachment.

Our results also reveal that extended work availability as a characteristic of boundaryless working hours that reflects increasing flexibility demands plays an important role for employees’ state of recovery. More specifically, the possibility and unpredictability of being contacted during free time for work-related reasons prevent recovery experiences, which in turn result in a poor state of recovery. Extended work ability disadvantageously affects not only psychological detachment but also relaxation and control. Therefore, an implication of our study is to avoid or minimize extended work availability whenever possible. Previous studies indicate that extended work availability can be affected by organizational culture and social norms in the workplace (e.g., Derks et al. 2015; Thörel et al. 2020). Thus, executives could adjust organizational culture and norms referring to extended work availability by communicating that they neither expect extended work availability nor equate it with high performance or commitment.

However, it is sometimes impossible for organizations to avoid overtime, extended work availability, or supplemental work at non-standard times, for instance, because of customer expectations, technical requirements, or order peaks. In such cases, organizations should enable their employees to recover sufficiently from their work directly after periods of boundaryless working hours, for example, by reducing their workload and dismounting their overtime hours using days off in lieu. In the long term, organizations should implement strategies to help employees to stay healthy even with boundaryless working hours. Since previous research has shown a buffering effect of boundary creation (Barber and Jenkins 2014), boundary management trainings (e.g., Michel et al. 2014; Rexroth et al. 2017) could be helpful interventions.

## Data Availability

Data of the BAuA-Working Time Survey are available as scientific use file at https://www.baua.de/DE/Aufgaben/Forschung/Forschungsdaten/Forschungsdaten_node.html. The SPSS-codes are available from the corresponding author upon reasonable request.
